# Emotion classification using a CNN_LSTM-based model for smooth emotional synchronization of the humanoid robot REN-XIN

**DOI:** 10.1371/journal.pone.0215216

**Published:** 2019-05-02

**Authors:** Ning Liu, Fuji Ren

**Affiliations:** Faculty of Engineering, Tokushima University, Tokushima, Japan; Politehnica University of Bucharest, ROMANIA

## Abstract

In this paper, we propose an Emotional Trigger System to impart an automatic emotion expression ability within the humanoid robot REN-XIN, in which the Emotional Trigger is an emotion classification model trained from our proposed Word Mover’s Distance(WMD) based algorithm. Due to the long time delay of the WMD-based Emotional Trigger System, we propose an enhanced Emotional Trigger System to enable a smooth interaction with the robot in which the Emotional Trigger is replaced by a conventional convolution neural network and a long short term memory network (CNN_LSTM)-based deep neural network. In our experiments, the CNN_LSTM based model only need 10 milliseconds or less to finish the classification without a decrease in accuracy, while the WMD-based model needed approximately 6-8 seconds to give a result. In this paper, the experiments are conducted based on the same sub-data sets of the Chinese emotional corpus(Ren_CECps) used in former WMD experiments: one comprises 50% data for training and 50% for testing(1v1 experiment), and the other comprises 80% data for training and 20% for testing(4v1 experiment). The experiments are conducted using WMD, CNN_LSTM, CNN and LSTM. The results show that CNN_LSTM obtains the best F1 score (0.35) in the 1v1 experiment and almost the same accuracy of F1 scores (0.366 vs 0.367) achieved by WMD in the 4v1 experiment. Finally, we present demonstration videos with the same scenario to show the performance of robot control driven by CNN_LSTM-based Emotional Trigger System and WMD-based Emotional Trigger System. To improve the comparison, total manual-control performance is also recorded.

## Introduction

Approximately 2100 years ago, King Mu of Chou made a tour of inspection in the west, on his return journey, a man named Yen Shih presented a handiwork which could sing, act and made the King think it was a real man in astonishment [1, 2]. Since that time, from East to West, making an automation that can mimic human activities has attracted great interest of talents such as the scholars of Alexandria, Leonardo da Vinci, Nikola Tesla and many modern scientists.

Year by year, the innovation of automation enables robot developments, and over decades of technology improvement, many humanoid robots have been developed for social use, such as WABOT by Waseda University, Asimo by Honda, Robonaut by NASA, Nadine by Nanyang Technological University and Actroid by Kokoro. Some of the humanoid robots have the same body structure as humans and can walk, hold objects, run and jump [3]. Some of the humanoid robots have human-like faces, and these robots can sing, speak languages and make facial expressions; one of them named Sophia developed by Hong Kong-based company Hanson Robotics, even become a Saudi Arabian citizen.

Despite the substantial progress achieved in the robot field, the expression controls of a humanoid robot are still manually operated by the developer for specified or limited scenarios. To extend the application scenes, one way to address this limitation is to annotate nine basic expression actions for our Actroid robot; regarding interaction, we utilized the proposed optimized WMD method, which can recognize nine emotion categories in texts [4] as an Emotional Trigger to generate the corresponding action labels according to the robot response. For the robot system, running the computed basic expression and the voice at the same time, we can obtain an acceptable humanoid robot interaction with emotional expression.

Due to the long time delay of the WMD algorithm, it takes approximately 5-10 seconds to calculate the emotion labels for every interaction loop. Thereby, for people who are communicating with a humanoid robot, the delayed feedback results in a loss of communication desire.

To address this gap, we propose a CNN_LSTM-based deep neural network model with thousands of times acceleration ability. In this paper, we utilize the same sub-data sets of the Chinese emotional corpus(Ren_CECps) used in former WMD experiments: one comprises 50% data for training and 50% for testing(1v1 experiment), and the other comprises 80% data for training and 20% for testing(4v1 experiment). The experiments are conducted with the WMD, CNN_LSTM, CNN and LSTM. The results show that CNN_LSTM gets the best F1 scores of 0.35 in the 1v1 experiment and almost the same accuracy as WMD with F1 scores of 0.367 to 0.366 in the 4v1 experiment. In the training process, our experiments show that the DNNs need only 3 epochs to train the models to readiness. This factor results in a difference between minutes and weeks cost in training and promises extended flexibility for the Actroid robot. The CNN_LSTM model is not entirely optimized, as it still has weaknesses, as discussed at the end of this paper.

The reminder of this paper is organized as follow: Section 2 presents related works. Section 3 introduces emotion-enhanced interaction for REN-XIN, in which the Emotional Trigger System, our actroid robot REN-XIN and the Ren_CECps corpus are described. Section 4 reviews the experiments and results. In Section 5, discussion is presented. Section 6 presents the conclusions and future works.

## Related works

Since robots were created, making them more engaged has become one of the predominant research fields. Marian et al. [5] deployed a real time facial expression system in the Aibo robot and the RoboVie robot to enhance user enjoyment. Diego et al.developed a framework to recognize human emotions through facial expressions for NAO [6]. To enable real-time facial expressions on humanoid robot REN-XIN, a forward kinematics model was proposed [7]. Since building a whole-length robot is expensive, some groups try to verify their facial expression system on the head only robot. K Berms and J Hirth [8] utilized 6 basic facial expressions for humanoid robot head ROMAN, which was a behavior-based control system. Hashimoto et al. developed a face robot for rich facial expression; this face robot has 18 control points and can easily imitate six typical facial expressions [9]. A quick application of facial expressions with head-neck coordination is employed on robot SHFR -III [10]. Another way to create expression emotions for a robot partner (in this example, iPhonoid-B) is to combine the facial and gestural expressions together, as implemented with a smart phone and servos [11]. However, all of the robots are controlled manually depending on limited scenarios.

Emotion computing, or sentiment analysis, is one of the most active research areas in the NLP field [12]. Most of the work has focused on social networks such as Twitter [13, 14], blogs [15, 16], microblog [17], or aimed to e-commerce purpose such as movie reviews [18], products opinions [19].

Limited to the scale of an annotated corpus, the previous studies have always relied on emotional-lexicon-based methods [20, 21]. The annotated emotion categories also vary according to the different corpus: from two(positive, negative) to four emotions(anger, fear, sadness and joy) [22] or eight emotions(anger, anxiety, expect, hate, joy, love, sorrow and surprise) [15]. These nonuniform annotated methods make this research field harder to extend to a large-scale corpus.

For decades, methods based on deep neural networks have achieved substantial progress. We can train a vector to present a word without labeled texts [23], and this ability perfectly solves the semantics loss in word representation for lack of standard data. A sentiment tree-bank proposed for semantic compositionality [17] moves the field into the deep learning era.

Many methods based on deep neural networks have been proposed, including a CNN-based neural network that can achieve state of the art results within little hyperparamter tuning [24], a neural network trained for a dialogue generation joining with emotion encoder [25], a simple LSTM model for affect recognition framework [26], and a combined model CNN-RNN applied to video emotion recognition [27]. For more refined word representation, Glove was proposed [28]. In addition, many new deep neural networks have been presented, such as GAN [29], ResNet [30] and a sequence to sequence model for emotional chatting [31].

## Emotion-enhanced interaction for REN-XIN

In this part, we introduce Emotional Trigger System, which is designed for emotion-enhanced interaction for Actroid REN-XIN. First, the robot platform REN-XIN and the corpus named Ren_CECps used in this paper are presented in the following subsection.

### Actroid and Ren_CECps

#### Actroid REN-XIN

**Fig 1:**
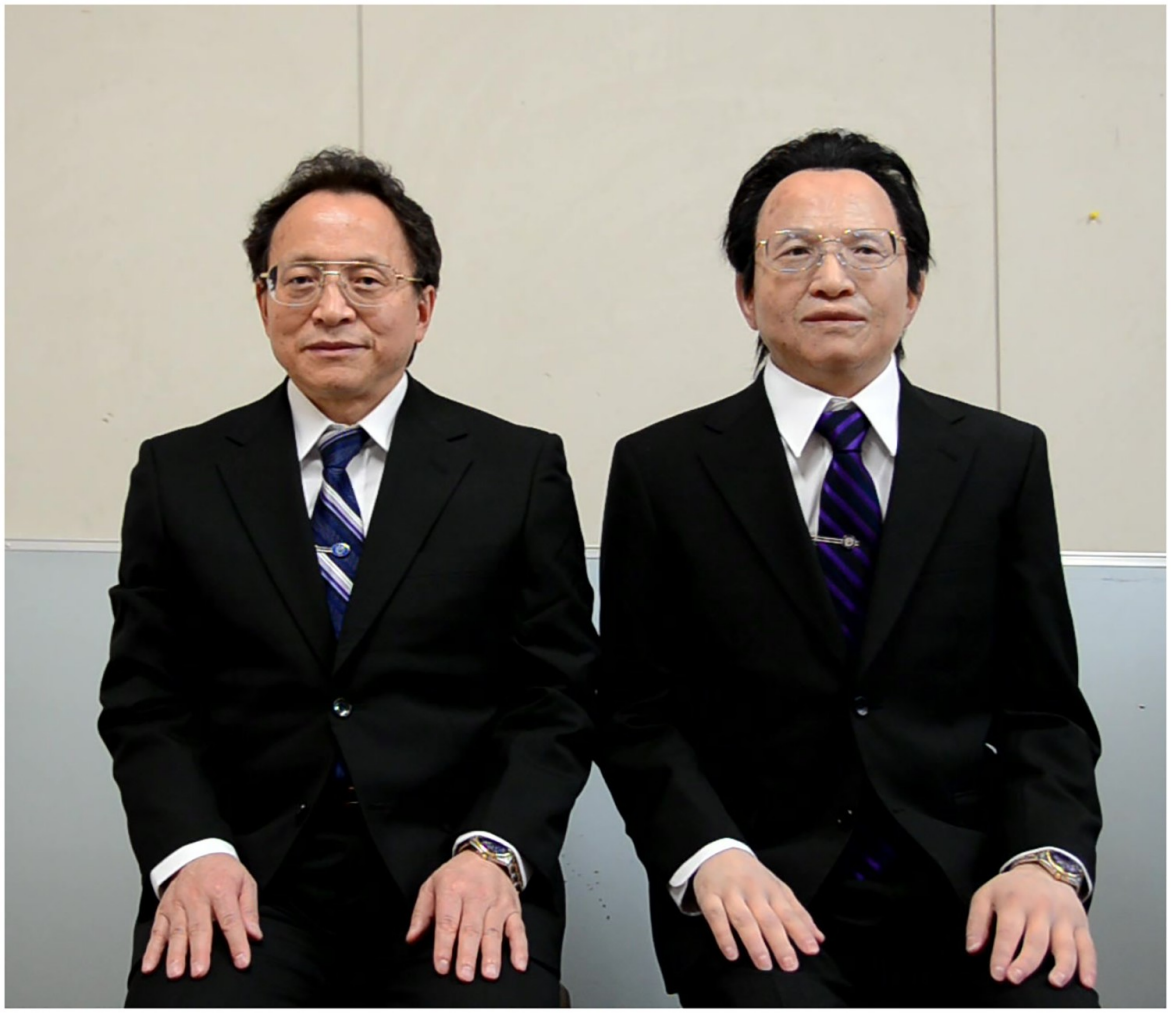
Prof. Ren (left) and his avatar robot REN-XIN (right).

Actroid REN-XIN is a humanoid robot developed by Kokoro Company Ltd. based on Prof. Ren with almost the same clothes and even the same spectacles worn by Prof. Ren as shown in Fig 1. The two eyes of REN-XIN are embedded in cameras, and to adjust the myopic lens, the two cameras are also modified with focusing ability.

REN-XIN has 12 degrees of freedom: seven for face control, three for the head and the last two for the upper body. Below are the detailed introductions for every degree and their control codes:

- eyebrow up or down;
  - scale, number ranges from 0 to 255, where 0 means the lowest eyebrow position and 255 means the highest position.
- cheek pulling;
  - scale, number ranges from 0 to 255, where 0 means no control on the cheek and with the apples of the cheeks not showing. In contrast, 255 means the highest position to pull the cheek; In this highest position, the apples of the cheeks are clearly shown.
- eyelids closed or opening;
  - scale, number ranges from 0 to 255, where 0 means the two eyelids are closed and 255 means that the eyelids are completely open.
- eyes turning right or left;
  - scale, number ranges from 0 to 255, where 0 means the rightmost position of eyes and 255 means the leftmost position of eyes.
- eyes moving up or down;
  - scale, number ranges from 0 to 255, where 0 means the lowest position to move the eyes down and 255 means the highest position to move the eyes up.
- mouth closed or opening;
  - scale, number ranges from 0 to 255, where 0 means closing the mouth and 255 means opening the mouth completely.
- mouth opening roundly or not;
  - scale, number ranges from 0 to 255, where 0 means no control operation and 255 means opening the mouth roundly at the largest parameter.
- left-side head tilt
  - scale, number ranges from 0 to 255, where 0 means no control operation and is the normal position and 255 means tilting the head to the leftmost position.
- right-side head tilt
  - scale, number ranges from 0 to 255, where 0 means no control operation and is the normal position and 255 means tilting the head to the rightmost position.
- head rotation
  - scale, number ranges from 0 to 255, where 0 means rotating the head to the rightmost angle and 255 means rotating the head to the leftmost angle.
- shrug or not
  - scale, number ranges from 0 to 255, where 0 means no shrug and 255 means shrugging the shoulders to the highest position.
- body leaning forward or backward
  - scale, number ranges from 0 to 255, where 0 means leaning the body to the most backward angle and 255 means leaning the body to the most forward angle.
- Because the servos are different from their initial position, not all of them start from the zero value, and in some spatial situations, the maximum value of 255 cannot be achieved.

**Fig 2:**
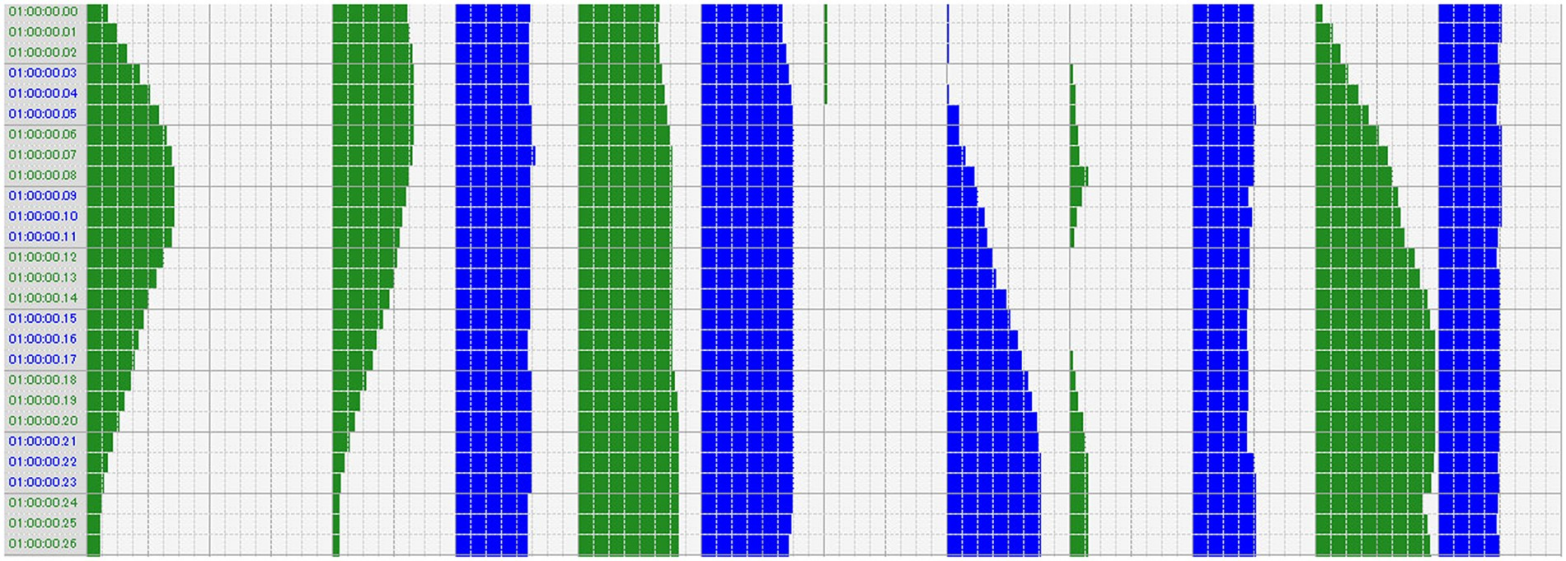
Sample fragment of the action encoder for REN-XIN.

The humanoid robot REN-XIN can be controlled by a movement GUI editor. Fig 2 shows a sample fragment of an action encoder with GUI for REN-XIN. Every row represents one movement per 1/60 s. In Fig 2, there are 98 columns per row, and every 8 columns represent the control code of corresponding servos ranging from 0 to 255. There are 12 ranges corresponding to the 12 degrees mentioned above.

In this sample, we have 26 movements; some servos are controlled by linear variation control codes, while some are discretely encoded. The colored histogram means the encoded value of corresponding servo, and the whitespace means 0.

#### Ren_CECps

Ren_CECps is the abbreviation of Chinese emotional corpus annotated by Ren Lab [15] at http://a1-www.is.tokushima-u.ac.jp/member/ren/Ren-CECps1.0/DocumentforRen-CECps1.0.html. This corpus contains 1487 blogs crawled through Internet. All blogs are annotated by the document→ paragraph→sentence structure.

**Fig 3:**
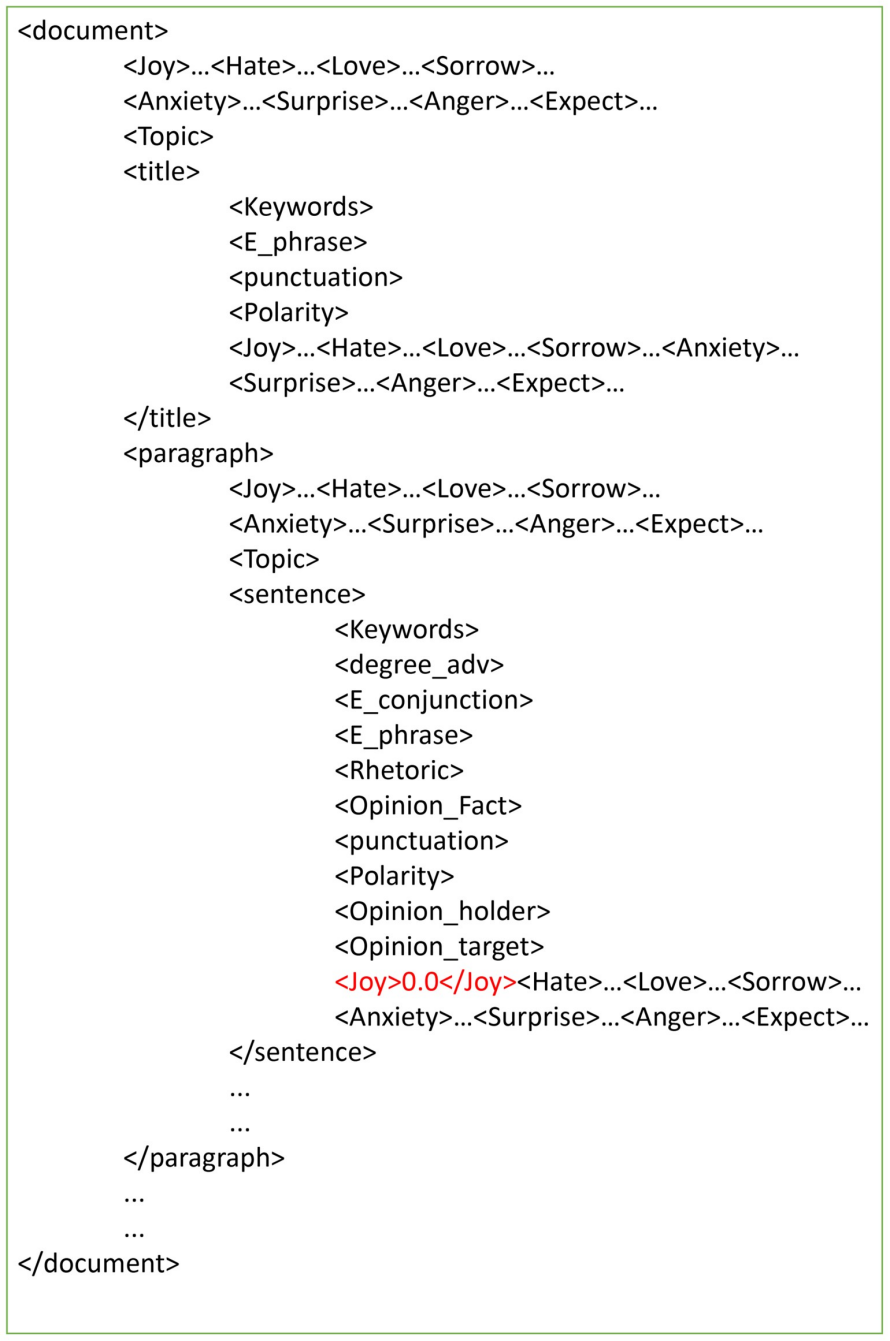
Sample of three-level annotation structure of Ren_CECps.

Fig 3 shows a sample of the document→ paragraph→sentence annotation structure of the blogs in Ren_CECps. As shown above, the three-level annotation structure can be clearly tagged by the < *document* > tag, < *paragraph* > tag and < *sentence* > tag. Each level is annotated with eight emotion categories (Joy, Hate, Love, Sorrow, Anxiety, Surprise, Anger, Expect) and its corresponding intensity (such as the red-tagged < *Joy* >). The intensity is a discrete value between 0.0 and 1.0. For the title of document, the polarity is also annotated. In each of < *document* > and < *paragraph* > levels, the topics are also targeted at the value of < *Topic* > tag following the eight emotion tags.

The sentence level is the most important level to annotate, as almost all emotions are contained in this level. The polarity and eight emotion categories of every sentence are annotated by default, and the emotional keywords and phrases are tagged in the tags of < *Keyword* > and < *E*_*phrase* > the same as the tags in < *title* >. Specifically, the eight emotion categories and corresponding intensities are annotated for word-based emotion research which are not shown in Fig 3. Some linguistics information is annotated to extend the use of this corpus, such as degree adverb tagged as < *degree* >, punctuation as < *punctuation* > and rhetoric as < *Rhetoric* >. For opinion mining, the opinion holder and opinion target are annotated with tags of < *Opinion*_*holder* > and < *Opinion*_*target* > respectively. In < *punctuation* > tag of title and sentence, the emotion type is added if appropriate.

In this paper, we utilize the same sub-data sets of Ren_CECps for our experiments used in the WMD experiments [4] which are split in two ways: one comprises 50% data for training and 50% for testing(1v1 experiment), and the other comprises 80% data for training and 20% for testing(4v1 experiment).

### Emotional Trigger System

**Fig 4:**
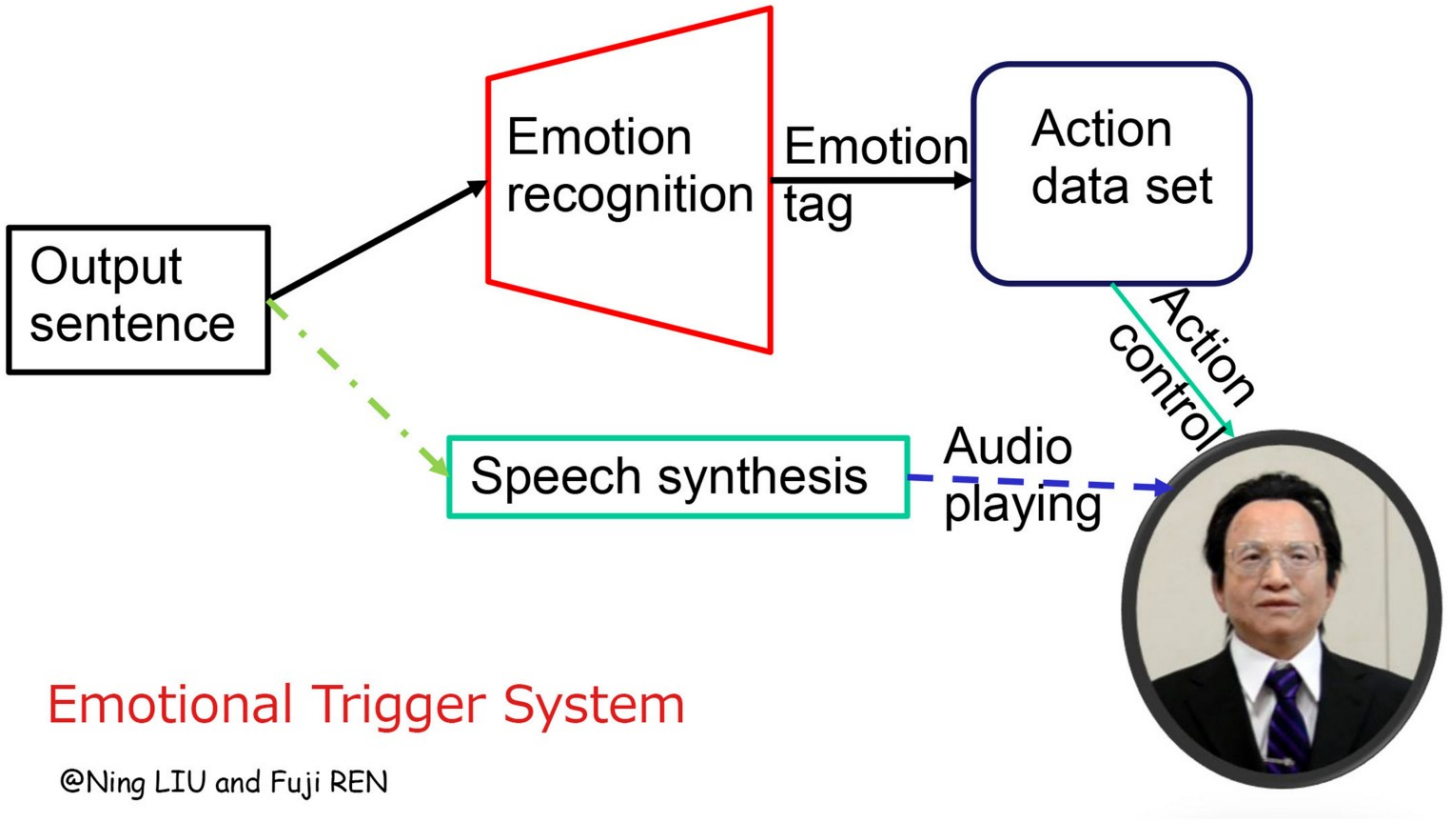
Flowchart of the Emotional Trigger System.

As mentioned above, almost all humanoid robots have to encode the facial actions manually, making it impossible to control the robot in real-time interaction with humans beyond the scripts. To give a solution for unknown situations, we design an Emotional Trigger System to give the humanoid robot REN-XIN the capability of automatic emotion expression. The system flowchart can be found in Fig 4.

As clearly shown in the figure, the system is designed to enhance the emotion expression of a humanoid robot in the response process: for the given answer content, two processes are established. One is speech synthesis, as a text-to-speech (TTS) service. The other one is an emotion recognition model(Emotional Trigger), in which the embedded classifier gives the response text an emotional label, and according to this computed label, the system can get the corresponding action code by searching the Action Database. Ultimately, running the action control model and audio playing model synchronously, we can have an “automatic” emotional interaction.

#### WMD based emotional trigger

Utilizing the built system as described above, we embed the WMD [4] model to recognize the emotion categories as the emotional action trigger during the interaction with REN-XIN. In this way, we need to encode only several emotional actions for our humanoid robot REN-XIN. As the WMD model was trained based on Ren_CECps with neutral emotion, to coordinate with the emotional action trigger, we need only to make eight emotional actions and a neutral face code. Actually, we edit 11 emotional movements: Anger, Anxiety, Expect, Fear, Hate, Joy, Love, Neutral, Sadness, Surprise, Tired. All of the actions can be found at http://a1-www.is.tokushima-u.ac.jp/ET/emoDB.html in the Humanoid Robot Emotional Movement DB.

With the encoded emotional actions, the Emotional Trigger System is ready to activate by the emotional triggers and enhances the interaction experience with humanoid robot REN-XIN.

#### DNN models for emotional triggers

According to our former three time-consuming experiments, the WMD needs 0.047s, 0.042s and 0.031s to finish ten computational replicates [4]. A fully computed input vector has 1800 dimensions, it’s a 1800 times’ computing based on the seed corpus. On average, we need 7.2 seconds to prepare the emotional trigger.

For real-time interaction, a seamless user experience is an essential aspect; thus the long time-delay of WMD-based Emotional Trigger must be addressed. The WMD has a best average time complexity of *O*(*N*^3^ log *N*) [32], where *N* denotes the vocabulary length. The average time complexity of deep neural network is *O*(*N*_*input*_ × *N*_*output*_ × *L*). (For filters in convolution neural network, the time complexity should consider the amount of filters. As the platforms used for deep neural network employ automatic parallel computing, in practice, we do not take the number of filters into consideration.) Here, *N*_*input*_ means the input vector dimension of one layer and *N*_*output*_ means the output vector dimension of one layer, *L* means the depth of the neural network. When dealing with natural language processing problems with small scale corpus, the depth of deep neural network is not always particularly deep, which means that *L* is much less than *N* and that an appropriate deep neural network will mitigate the time gap.

**Fig 5:**
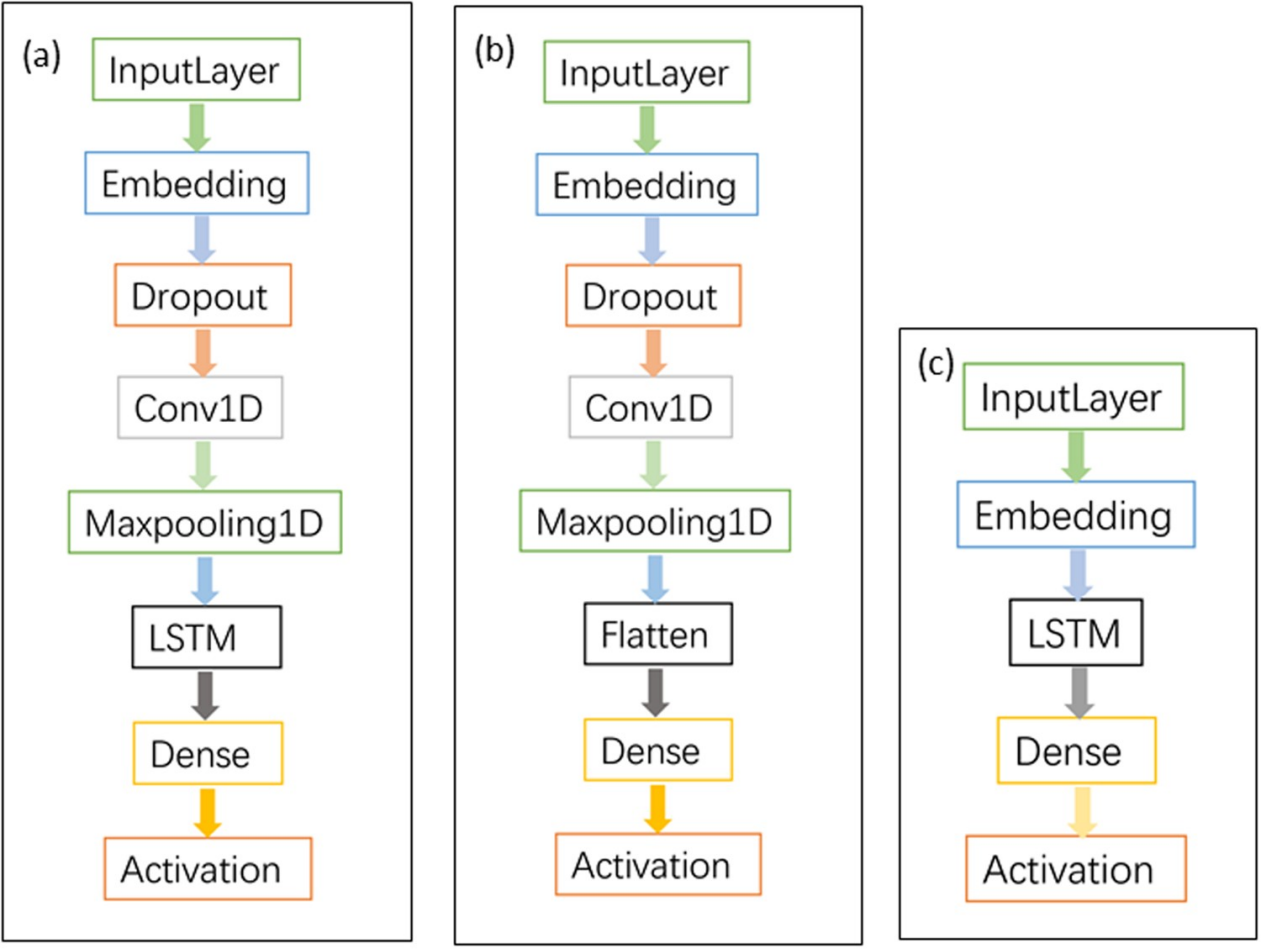
Structure of the three neural networks. (a) CNN_LSTM-based network. (b) CNN-based network. (c) LSTM-based network.

In this paper, we propose a CNN_LSTM-based network as the emotional trigger to give a better interaction experience. For a full comparison, CNN- and LSTM-based networks are also designed in the experiments, all of the codes can be found at https://github.com/colorcatliu/ETSystem_model. The three deep neural networks are shown in Fig 5, and the introduction of structures is as follows:

**CNN_LSTM-based structure** The CNN_LSTM-based structure has proven to achieve excellent performance on sentiment classification [33]. In this paper, we construct the traditional layers to combine the speed of CNN and the temporal semantics of LSTM together. As shown in Fig 5(a). Input layer is followed by an embedding layer to train a sentence vector for the next CNN layer.
**CNN-based structure** The CNN-based structure is a simple convolution network in which the “Flatten” layer takes place of the “LSTM” layer. The connection layers are described in detail in Fig 5(b).
**LSTM-based structure** The LSTM-based network is much lighter than the two networks above. One Embedding layer followed by a LSTM layer and then a Dense layer for outputting the emotional triggers. Fig 5(c) shows the architecture.

## Experiments and results

The experiments proceed in three parts: The first is to verify the response times of Emotional Triggers based on WMD, CNN_LSTM, CNN and LSTM; The second is to apply the classification performance tests with standard micro-F1 and macro-F1 scores based on Ren_CECps; And the third part is a real time demonstration, in which we choose WMD and the best model among the three networks to build two Emotional Trigger Systems. The emotion expressions of humanoid robot REN-XIN based on the two constructed Emotional Trigger Systems are characterized, and one manually encoded emotion expression in the same scenario is presented as a comparison experiment.

### Setup

In this paper, we verify the classification abilities among WMD, CNN_LSTM-based network, CNN-based network and LSTM-based network using the split 1v1 and 4v1 data sets of Ren_CECps. The response time experiments among these algorithms follow the same ten-time computing rule. We randomly select 30 sentences from Ren_CECps, manually collecting the first 10, second 10 and the last 10 sentences as three groups of the ten-replicate computing experiment. The seed corpus needed for WMD is the same as that of previous experiments.

To make a sententious declaration for the experiments above, we give the abbreviations as follows:

- **WMD_1v1**: the WMD method based on the 1v1 data set;
- **WMD_4v1**: the WMD method based on the 4v1 data set;
- **cnn_lstm_1v1**: the CNN_LSTM network based on the 1v1 data set;
- **cnn_lstm_4v1**: the CNN_LSTM network based on the 4v1 data set;
- **cnn_1v1**: the CNN network based on the 1v1 data set;
- **cnn_4v1**: the CNN network based on the 4v1 data set;
- **lstm_1v1**: the LSTM network based on the 1v1 data set;
- **lstm_4v1**: the LSTM network based on the 4v1 data set;

### Hyperparameters

In the experiments, we use Keras [34] as the construction API for the DNNS and the backend is TensorFlow [35]. All of the sentences are preprocessed into sequences with the words presented by their indexes in the lexicon. The vocabulary length is 32,126. Table 1 shows the lengths of sentences in Ren_CECps. The statistics show that more than 99.9% of the sentences have a length of less than 200; thus, the “maxlen” of padding is selected as 200.

**Table 1:** Lengths of sentences in Ren_CECps.

length	total	0-200	200-300	300-500
sentence no.	50321	50312	7	2
per. (%)	100	99.982	0.0139	0.0041

Limited by the scale of the corpus, embedding size is set to 128, 0.5 for the dropout percentage. For the convolution layer, kernel size is 5, calculated with 64 filters. The corresponding MaxPooling layer gets 4 as the pool size. We make the output size of LSTM layer as 70, and for batch size, 30 is selected. All of the parameters are chosen empirically.

Other parameters for Conv1D layer are “padding” with “valid”, “activation” with “relu” and “strides” with “1”. For the last dense layer, we make it a nine cells with “RandomUniform” as “kernel_initializer” corresponding to the loss parameter of “categorical_crossentropy” on nine emotion categories. The activation function for dense layer is “softmax”. We use “adam” to optimize loss function and “accuracy” for metrics of the network. The number of epochs is 3 exported through experiments, as explained in the discussion section. All of the codes can be accessed at https://github.com/colorcatliu/ETSystem_model.

### Evaluation measures

In this paper, we use both the micro standard and macro standard for calculating the precision and recall. The evaluation is measured by F1-score:

$$F1=\frac{2*precision*recall}{precision+recall}$$ (1)

where:

$$\begin{array}{c} precision=\{\begin{array}{cc} Mean(\sum \frac{tp_{i}}{tp_{i}+fp_{i}}) & ,\;if\;macro-average\\ \frac{tp}{tp+fp} & ,\;if\;micro-average \end{array}\\ recall=\{\begin{array}{cc} Mean(\sum \frac{tp_{i}}{tp_{i}+fn_{i}}) & ,\;if\;macro-average\\ \frac{tp}{tp+fn} & ,\;if\;micro-average \end{array} \end{array}$$

Here, *tp* is the number of global true positives, *fp* is the number of global false positives and *fn* is the number of global false negatives when using the micro standard; *tp*_*i*_ is the number of true positives of category *i*, *fp*_*i*_ is the number of false positives of category *i* and *fn*_*i*_ is the number of false negatives of category *i* when using the macro standard. In this paper, both of *precision* and *recall* are calculated in ‘macro’ and ‘micro’ model using the *metrics* package in sklearn [36].

### Response time and classification results

**Fig 6:**
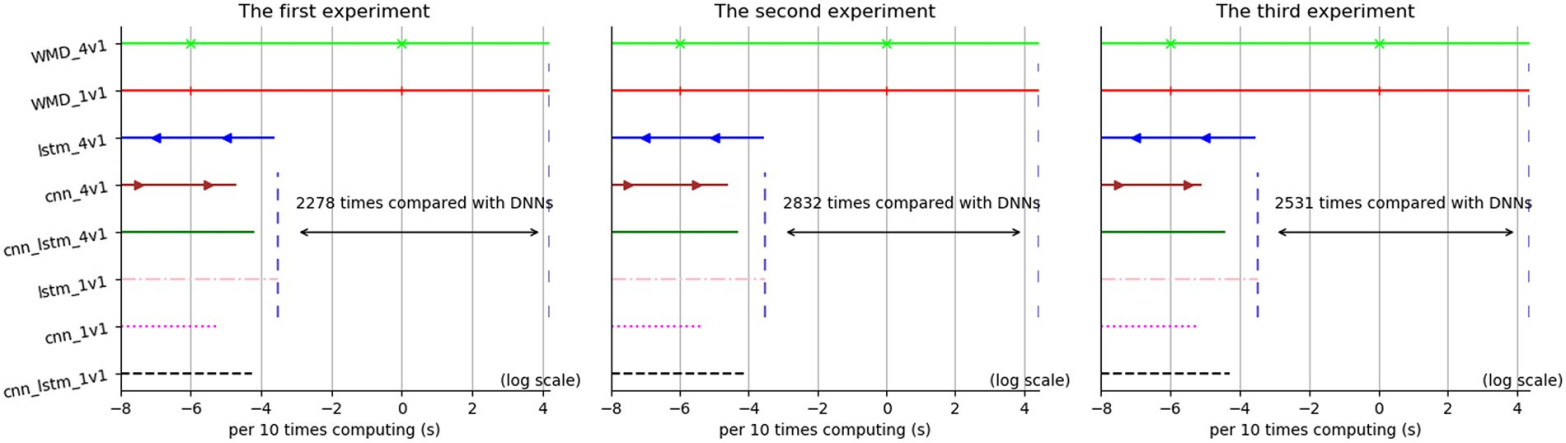
Acceleration results among WMD and the three networks.

**Fig 7:**
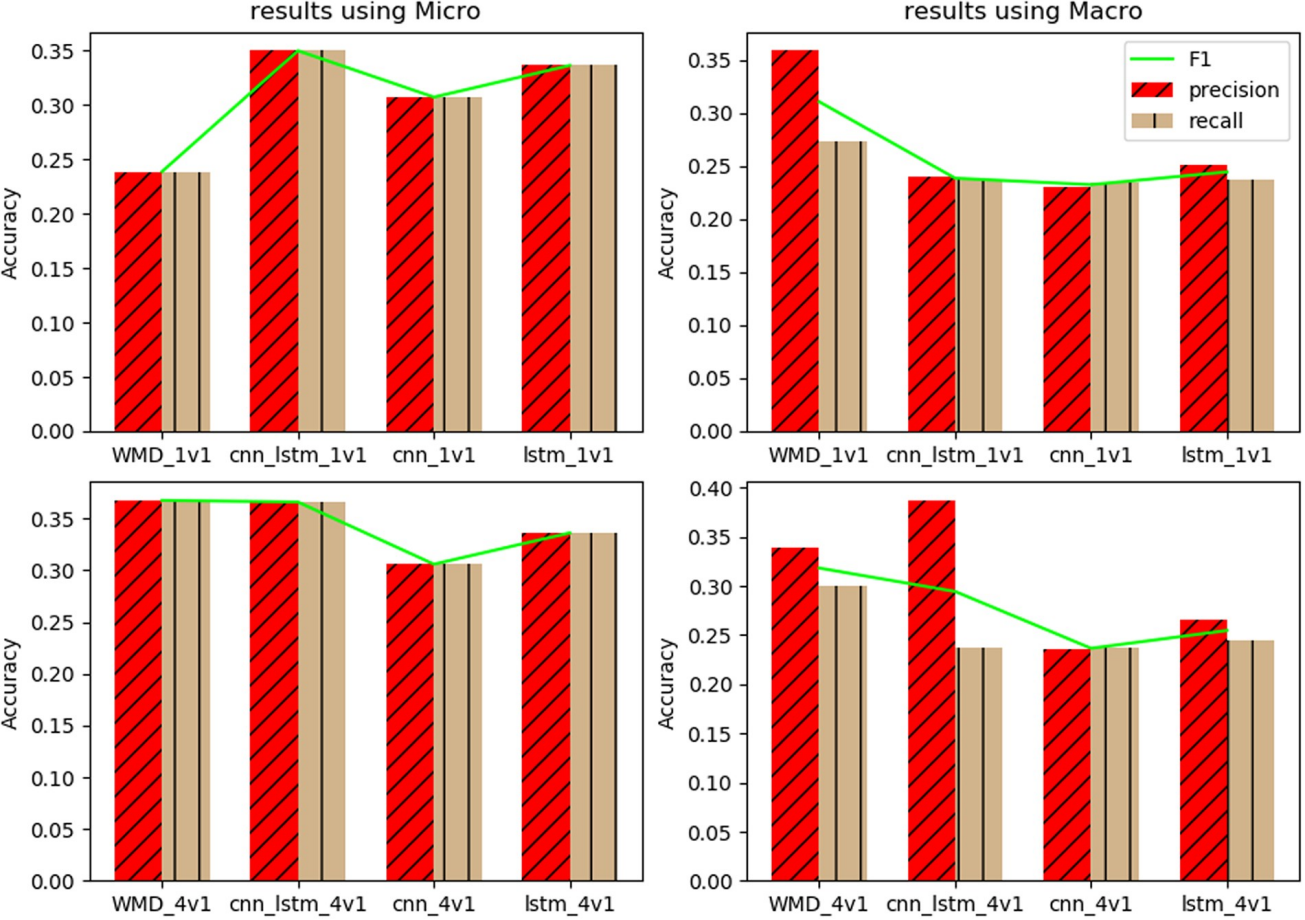
Classification results on WMD and the three networks.

Relying on the parameters and corpus, we can get the time consumption results of WMD and the three networks in Table 2. Fig 6 shows the acceleration lines with the values shown on a log scale. Table 3 gives the precision, recall and F1-score of WMD and three networks over the “micro” and “macro” standards, and Fig 7 shows the tendency graph based on these data.

**Table 2:** Time consumption results of WMD and the three networks.

Methods	Group 1	Group 2	Group 3	Average	Median	Standard Deviance
	per 10 times(s)					
**WMD_1v1**	**66.26106**	84.76050	**77.94079**	76.32078	77.94079	7.63874
**WMD_4v1**	66.26100	**86.76047**	77.94075	76.98740	77.94075	8.39597
**cnn_lstm_1v1**	0.01401	0.01770	0.01348	0.01506	0.01401	0.00187
**cnn_1v1**	**0.00557**	**0.00495**	**0.00565**	0.00539	0.00557	0.00031
**lstm_1v1**	0.02908	0.02992	0.03078	0.02992	0.02992	0.00069
**cnn_lstm_4v1**	0.01458	0.01292	0.01167	0.01305	0.01292	0.00119
**cnn_4v1**	0.00866	0.00975	0.00588	0.00809	0.00866	0.00162
**lstm_4v1**	0.02605	0.02784	0.02817	0.02735	0.02784	0.00093

**Table 3:** Classification results on WMD and the three networks.

Type	Algorithm	Precision	Recall	F1-score
**Micro**	WMD_1v1	0.23887	0.23887	0.23887
	cnn_lstm_1v1	0.35002	0.35002	**0.35002**
	cnn_1v1	0.30734	0.30734	0.30734
	lstm_1v1	0.33647	0.33647	0.33647
	WMD_4v1	0.36759	0.36759	**0.36759**
	cnn_lstm_4v1	0.36600	0.36600	**0.36600**
	cnn_4v1	0.30596	0.30596	0.30596
	lstm_4v1	0.33618	0.33618	0.33618
**Macro**	WMD_1v1	0.35858	0.27378	**0.31050**
	cnn_lstm_1v1	0.23938	0.23752	0.23844
	cnn_1v1	0.23065	0.23413	0.23237
	lstm_1v1	0.25158	0.23760	0.24439
	WMD_4v1	0.33847	0.30025	**0.31822**
	cnn_lstm_4v1	**0.38705**	0.23755	**0.29441**
	cnn_4v1	0.23624	0.23696	0.23660
	lstm_4v1	0.26606	0.24427	0.25470

In Table 2, we clearly shows a thousandfold acceleration after incorporating deep neural networks. For an average time in one ten-replicate computation, the WMD needs 76 s, that is 7 s per recognition, the same as with our previous experiments. The LSTM-based model needs more time than the CNN- and CNN_LSTM-based models: 0.03 s in total and 3 milliseconds per recognition on average. In Fig 6, we draw the slate blue guide line as the lower limit based on the LSTM model and the upper limit based on WMD. The results correspond to a maximum promotion factor of almost 3000. In these results, the CNN-, LSTM- and CNN_LSTM-based networks provide high-efficiency to solve the time delay problem.

On the other hand, although the DNNs obtain the fastest emotional trigger, without higher or at least similar classification ability, the speed is useless. Table 3 and Fig 7 show the verification experiments for this issue. In Fig 7, we draw two histograms to represent precision and recall separately, in which a red color with a slash texture means precision, tan with a vertical texture denotes recall and F1 scores are drafted as polylines in lime. The results of 1v1 and 4v1 are placed together by 4 sub-figures, the first row represents 1v1 experiments, and the second row represents 4v1 experiments.

In the experiments, we measure the results based on the Micro and Macro standards. Considering the Micro standard, in the 1v1 experiments, CNN_LSTM gets the best F1 score of 0.35002. All of the three networks obtain F1 scores over 30% and 12 percentage points higher than the WMD model. With the additional training data in the 4v1 experiments, WMD obtains the best F1 score of 0.367, a promotion of 13 percentage points from its 1v1 results and 0.1 percentage point higher than the best F1 score of 0.366 achieved by the CNN_LSTM model. With more training data, the WMD model exhibits a substantial promotion, while the CNN and LSTM models do not change. Only the CNN_LSTM network moves forward.

In Macro standard, however, the results are opposite. Fig 7 shows that WMD receives the best F1 scores in both 1v1 and 4v1 experiments. In the 1v1 experiment, the three networks get almost the same F1 scores, 7 percentage points lower than the WMD. In the 4v1 experiments, although WMD still receives the best F1 score, its promotion is only 0.8 percentage. While the CNN model exhibits the least improvement of 0.4%, the LSTM model is promoted by one percentage and the CNN_LSTM model achieves the greatest improvement of 7%. In this experiment, the CNN_LSTM network gets the best precision (0.387) among all of the algorithms.

Considering the Micro and Macro results, we find that in both calculation standards, the F1 scores of CNN_LSTM method are prominently improved from 1v1 to 4v1. WMD changes clearly only in the Micro experiments. The LSTM netowrk makes a small step only in Macro, and the CNN-based network keeps almost the same results from 1v1 to 4v1 in Micro and Macro.

In the results of the response time experiments and classification comparisons, the CNN_LSTM network proves its ability to impart a real time emotional trigger with acceptable accuracy relative to WMD. Thus, Actroid REN-XIN can make an emotional action with negligible time delay over the whole recognition and response processes when accommodating several scenes.

### Results of real time demonstration

According to the classification results described above, we choose the CNN_LSTM-based neural network and the WMD-based algorithm as two Emotional Triggers. To verify the performance of automatic emotion expression, we utilize an introduction script with manually encoded movements on the humanoid robot REN-XIN as baseline.

Table 4 shows the sentences of the script and the classified labels computed by the WMD- and CNN_LSTM-based Emotional Triggers. The experiment videos can be found at http://a1-www.is.tokushima-u.ac.jp/ET/et.html. In the project homepage, the “Original” video stands for the introduction demo of REN-XIN, (a) and (c) represent the introduction demo videos driven by the WMD-based Emotional Trigger Systems, (b) and (d) represent the introduction demo videos driven by CNN_LSTM-based Emotional Trigger Systems.

**Table 4:** Script used in real time demonstration.

Sentences	Results of Emotional Trigger	
	WMD	CNN_LSTM
好的, 我是任教授的化身, 我的名字叫任心	love	love
Ok, I am the avatar of Pro. Fuji Ren, my name is Ren Xin		
诞生于ニ零一二年十月十日日本德岛大学任研究室	neutral	neutral
I was born in Ren Lab, Tokushima University, Japan on Oct. 10, 2012		
我目前会说三种语言中文、日文、英文	neutral	love
I can speak three languages: Chinese, Japanese, English		
我是很富有情感的	love	love
I am very sensitive		
我现在可以表示八种情感	joy	love
I can express 8 emotions now		
包括高兴、悲伤、生气、厌恶	joy	love
Including happy, sad, anger, disgust		
惊讶、害怕、疲惫、平箭	anxiety	anxiety
Surprise, fear, tired, calm		
我给大家展现ー下吧	love	joy
Let me show it for you		
一般情况下, 我都会是这种平静的表情	joy	love
Usually I have this calm expression		
如果遇到个聪明伶倒的对象, 我就会很高兴	joy	joy
If I meet someone who is smart, I would be very happy		
遇到地震, 我也会害怕	anxiety	sorrow
I would be afraid in an earthquake		
別人说我很笨时, 我就会很悲伤	sorrow	sorrow
I would be very sad to be considered stupid		
遇到不平之事, 还会生气	anxiety	anxiety
If I meet the unfairness, I will be very anger		
如果你不尊重我, 我就讨厌你	love	hate
I disgust people who don’t respect me		
我疲惫的时候也需要休息, 这时你最好不要打扰我啦	expect	expect
I also need a rest if I’m tired, you’d better leave me alone at this time		
有意思吧, 你不要感到惊讶	expect	expect
Aha, it sounds interesting, don’t be surprise with my performance		
我目前还被认为是机器人	neutral	anxiety
I was identified as a robot currently		
但不久我就要加入到人类的行列了	anxiety	anxiety
But I will join the human society soon		
我的同类还没有谈过恋愛结过婚	neutral	sorrow
Our robots don’t have the experience of dating and marriage		
我有信心改变这种状况	anxiety	love
I have the confidence to change that		
我希望与人类共生	expect	expect
I would like to live together with human		
感谢您的厚愛	love	love
Thank you		

To verify whether real time text-to-speech model has an influence on the response time of the system, we make two different speech synthesis models: (a) and (b) use synthesized voices prepared before experiments, while (c) and (d) employ real time speech synthesis running with the Emotional Trigger Systems. For better viewing, the time axes of the four videos are all calibrated to eliminate the time delay before the first action.

The result videos show us that the Emotional Trigger System is efficient to give the robot an automatic emotion expression ability. Subjectively, the CNN_LSTM-based Emotional Trigger System has better interaction experience than the WMD-based system. In addition, the real time speech synthesis model performs as well as the local voice.

## Discussion

The proposed CNN_LSTM network successfully solves the long time delay with trainable accuracy; however there still are some key points to be discussed to characterize the experience for others or for related field research work.

### Epochs

**Fig 8:**
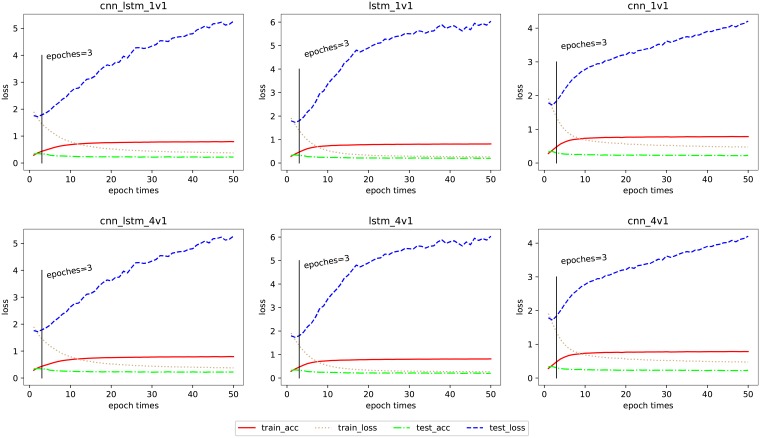
Tendency of accuracy and loss changed with the epochs in three networks.

Regarding the parameter of epochs, we run a 50-epoch experiment using the 1v1 and 4v1 data sets among all the three networks. The tendency of accuracy and loss changing with epochs is shown in Fig 8. The red line denotes the accuracy of the training data, the tan dotted line shows the loss calculated through training, the lime line represents the testing accuracy, and the blue dotted line is the corresponding loss.

In Fig 8, we annotate the guide lines of *epochs* = 3 in every sub-figures. In the model training process, the epochs of the three networks are all set to 3 according to this guide line, which is not an empirical selection. We can check the accuracy and loss pairs in every sub-figures, and the inflection point can be found in *epochs* = 2. All six sub-figures show the increase in training accuracies with the decrease in training loss epoch by epoch, while the test accuracies keep declining with test loss continuing to increase until there are no changes, unfortunately.

In our experience, the *epochs* = 2 should be the suitable parameter for training. With the contrary tendencies obtained from the six graphs, we make an assumption that the former experiences may not fit this multi-class emotional corpus well. To verify this hypothesis, we save the models after every epoch during our 50-replicate iteration and then calculate the F1 scores in Macro standard over every exported epoch-based model (the metrics parameters in Keras are all measured by the Micro standard). The results show that *epochs* = 2 obtains the best precision in Micro standard which is shown clearly in Fig 8, while *epochs* = 3 gets the best F1 scores. In the Micro standard, *epochs* = 3 obtains a better precision and F1 score than other epochs. According to these comparative trials, we finally set *epochs* as 3.

### Different promotion among the four methods

Table 3 and Fig 7 show that WMD performs well in Macro standard, whereas the CNN_LSTM method performs better in Micro. The results among the three networks also show different tendencies which is interesting.

We start at the beginning: the data split in Ren_CECps. The sentences are all annotated with 8 emotional labels mentioned before. If the labels are not annotated, the intensity values of corresponding categories are 0, and we record the category of these sentences as “neutral”. In the process of splitting data into a single category level, we add the sentence to the specific emotion if its intensity value is not 0, which means that one sentence can appear in the sub-data sets of more than one category; on the other side, the “neutral” sentences can appear in only one sub-data set.

In Ren_CECps, there are 36,525 sentences, 22,751 sentences have only one emotional category, 11,731 sentences have two emotional labels and 1,847 sentences have three labels. The 1v1 and 4v1 data sets are randomly selected based on the sub-data sets. Sentences with multi-label may appear multi-time in training and testing data with different classes. Under this framework, we can describe our experiments as multi-class classification with adversarial samples.

We first analyze the WMD method with the CNN_LSTM network. The contrasting tendencies in Micro and Macro results show the different feature representation abilities. In the adversarial data set, the WMD calculates the features from words to words with the distance measurement, which is better than sparse cells learning in the CNN_LSTM network, and results in an F1-score of 0.31 vs 0.23 in 1v1 and 0.31 vs 0.29 in 4v1 in Macro standard. In Micro, the CNN_LSTM model shows a stronger ability to learn global features than WMD, which results in a 10 percentage points acceleration of F1-score of 0.35 vs 0.23 in the 1v1 experiments. With more data, the WMD achieves a 0.1% higher F1 score than CNN_LSTM (0.367 vs 0.366), and the F1 score of WMD changes from 0.23 to 0.36. These results show that WMD can learn more global features than CNN_LSTM against more adversarial samples even though the scale of training data is also increased. The minimal improvement of CNN_LSTM shows that the network is sensitive with adversarial samples and cannot learn more global features. Returning to the Macro, the stronger ability of feature representation of WMD cannot achieve more promotion either, while CNN_LSTM gets a 6 percentage points increase. Combining the Micro and Macro results, we find that with additional training data, the CNN_LSTM network cannot learn global features, but the ability to learn local feature of categories still works and achieves prominent promotion. With more training data, the CNN_LSTM network can learn both global and local features better than the WMD method.

Next, we analyze the three networks. The almost unaltered scores in Micro show that the three networks are all sensitive and cannot learn more global features from more data with adversarial samples. In Macro, we can find in Table 3 that for the 1v1 to 4v1 experiments, the CNN-based network achieves almost the same scores of 0.232 to 0.236, and the LSTM-based network achieves a percentage point improvement of 0.244 to 0.254. The different promotions achieved between the CNN and LSTM networks provide a demonstration that the LSTM layer has a stronger ability than CNN to learn local features of categories against adversarial samples. This result emphasizes the ability of strong sequence information learning. However, the results do not indicate the failure of CNN; in CNN_LSTM, the achievement of a 6 percentage points improvement and the best results among three networks show that CNN can filter information of adversarial samples and enhance the learning ability of LSTM, thus resulting in the better learning ability of local features than WMD.

## Conclusion and future work

According to the comparison experiments and discussion, we can make the following conclusions:

1. We proposed an Emotional Trigger System which can extend the interaction of the humanoid robot REN-XIN for more emotional actions;
2. CNN_LSTM can considerably speed up the Emotional Trigger System without emotion classification accuracy descent compared with the WMD-based Emotional Trigger System;
3. The CNN_LSTM-based network has a stronger ability to train models against adversarial samples than networks based on only CNN or LSTM;
4. For training a small corpus using deep neural networks, the inflection point of training and testing is not always the best parameter, the next epoch may be better.

The CNN_LSTM model shows the strong ability to learn global features for multi-class corpus, although the local features do not perform well as expected. In the future, we will focus on training the networks for each emotional category separately without adversarial samples. For Actroid REN-XIN, we need more research to provide the robot with better auto-response ability without manually operated controls of emotional expression.
